# Gsk3**β** regulates the resolution of liver ischemia/reperfusion injury via MerTK

**DOI:** 10.1172/jci.insight.151819

**Published:** 2023-01-10

**Authors:** Hanwen Zhang, Ming Ni, Han Wang, Jing Zhang, Dan Jin, Ronald W. Busuttil, Jerzy W. Kupiec-Weglinski, Wei Li, Xuehao Wang, Yuan Zhai

**Affiliations:** 1 Dumont-UCLA Transplant Center, Department of Surgery, David Geffen School of Medicine, UCLA, Los Angeles, California, USA.; 2 Department of Hepatobiliary-Pancreatic Surgery, China-Japan Union Hospital of Jilin University, Changchun, China.; 3 Hepatobiliary Center, Key Laboratory of Liver Transplantation of Chinese Academy of Medical Sciences, The First Affiliated Hospital of Nanjing Medical University, Nanjing, China.; 4 Department of Obstetrics and Gynecology, Renji Hospital, School of Medicine, Shanghai Jiao Tong University, Shanghai, China.; 5 Transplant Surgery, College of Medicine, Medical University of South Carolina, Charleston, South Carolina, USA.

**Keywords:** Hepatology, Immunology, Innate immunity, Macrophages

## Abstract

Although glycogen synthase kinase β (Gsk3β) has been shown to regulate tissue inflammation, whether and how it regulates inflammation resolution versus inflammation activation is unclear. In a murine liver, partial warm ischemia/reperfusion injury (IRI) model, we found that Gsk3β inhibitory phosphorylation increased at both the early-activation and late-resolution stages of the disease. Myeloid Gsk3β deficiency not only alleviated liver injuries, it also facilitated the restoration of liver homeostasis. Depletion of Kupffer cells prior to the onset of liver ischemia diminished the differences between the WT and Gsk3β-KO mice in the activation of liver IRI. However, the resolution of liver IRI remained accelerated in Gsk3β-KO mice. In CD11b-DTR mice, Gsk3β-deficient BM-derived macrophages (BMMs) facilitated the resolution of liver IRI as compared with WT cells. Furthermore, Gsk3β deficiency promoted the reparative phenotype differentiation in vivo in liver-infiltrating macrophages and in vitro in BMMs. Gsk3 pharmacological inhibition promoted the resolution of liver IRI in WT, but not myeloid MerTK-deficient, mice. Thus, Gsk3β regulates liver IRI at both activation and resolution stages of the disease. Gsk3 inactivation enhances the proresolving function of liver-infiltrating macrophages in an MerTK-dependent manner.

## Introduction

Liver ischemia/reperfusion injury (IRI) is an inevitable pathological consequence of multiple clinical conditions, including hepatic tumor resection, transplantation, and trauma. An innate, immune-dominated inflammatory response drives the pathogenesis of liver IRI (1, 2). The danger-associated molecular pattern (DAMP) activates innate immune cells via pattern recognition receptors (e.g., TLR4, TLR9), leading to inflammatory liver injuries (3–7). Liver-resident Kupffer cells (KCs) represent approximately 35% of liver nonparenchymal cells (NPCs) and 80%–90% of all tissue macrophages in the entire body. They are the first responder to ischemia/reperfusion–induced hepatocellular damage by producing inflammatory cytokines or chemokines, leading to the infiltration and activation of peripheral monocytes and amplification of liver inflammation and injuries. The clinical outcome of liver IRI depends not only on the activation but also the resolution of the inflammatory response. Much less is known about the resolution of liver IRI. The involvement of KCs and infiltrating macrophages (iMΦs) in the restoration of liver homeostasis after IRI remains to be delineated.

Glycogen synthase kinase 3 (Gsk3) regulates multiple cellular functions, including metabolism, proliferation, differentiation, apoptosis, and immune activation, in different types of cells (8–14). The 2 isoforms of Gsk3, α and β, share extensive homology in the kinase domain but are functionally distinctive due to their diverse N and C terminals. Global Gsk3β-KO mice are embryonic lethal because of liver degeneration (15), whereas Gsk3α-KO mice are viable and normal (16). Gsk3 is unique in that it is constitutively active and inhibited upon stimulation by N-terminal phosphorylation (17). We have been interested in this kinase as a therapeutic target of liver IRI, in part because of its differential regulation of proinflammatory and antiinflammatory gene programs downstream of TLRs (9, 18). Importantly, pharmacological Gsk3 inhibitors have been developed and tested in neurological and metabolic diseases and cancer (19). Gsk3 inhibition downregulates TNF-α but upregulates IL-10 production in macrophages and DCs upon TLR stimulation (9, 18). In vivo, Gsk3 inhibitors protect mice from endotoxin shock (18). We have shown that Gsk3 inhibition protects mice from liver IRI via an IL-10–mediated immune regulatory mechanism (20) and that myeloid Gsk3β deficiency promotes regulatory macrophage differentiation via the AMPK-SHP–mediated pathway (21). More recently, we demonstrated that Gsk3α, but not Gsk3β, regulates hepatocyte autophagy in response to inflammatory stimuli, and its S21A mutant protects livers from IRI (22).

In the present study, we analyzed how Gsk3β regulates the restoration of liver homeostasis after ischemia/reperfusion (IR) in the murine-liver partial warm-ischemia model (23). By extending the reperfusion time from 6 hours to 7 days, we compared myeloid Gsk3β-deficient and WT mice in their resolution kinetics of tissue inflammation, focusing on the macrophage heterogenicity (KCs and iMΦs) and disease stages (activation and resolution). By selective depletion of KCs and reconstitution with BM-derived macrophages (BMMs) in CD11b-DTR mice, we demonstrate that Gsk3β plays critical roles in the resolution of liver IRI by controlling the development of immune regulatory and tissue reparative functions in iMΦs in an MerTK-dependent manner.

## Results

### Myeloid Gsk3β regulates liver inflammation resolution in IRI.

**Figure 1:**
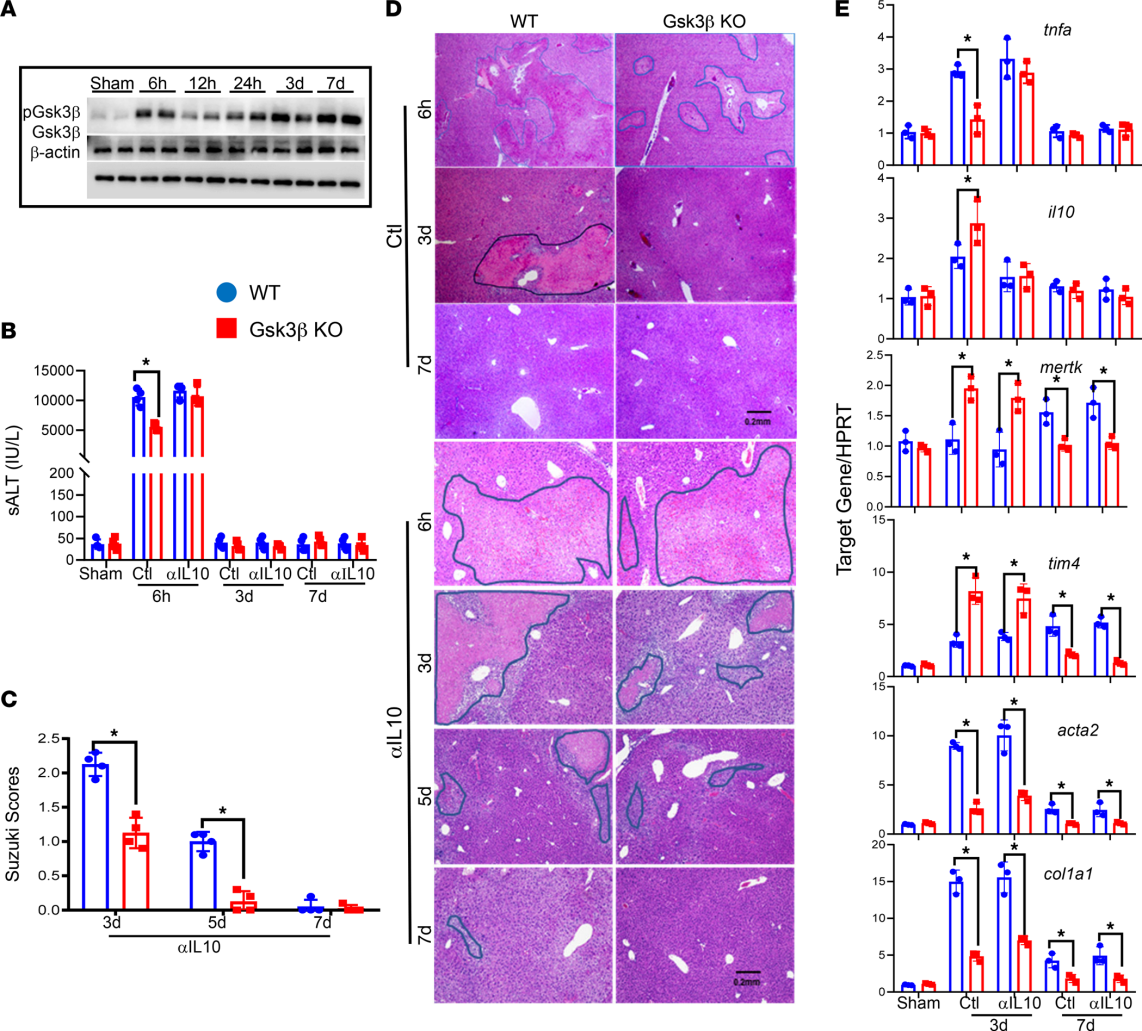
Gsk3β N-terminal phosphorylation and its regulation of the resolution of liver IRI. Myeloid Gsk3β WT and Gsk3β-KO mice were treated with control (Ctl) Ig or anti–IL-10 Ab 1 hour prior to the start of liver ischemia. Serum and liver tissues were harvested at various times after reperfusion, as described in Methods. (**A**) Western blots of total and S9-phosphorylated Gsk3β and β-actin in sham and IR livers of WT B6 mice at 6 hours, 12 hours, 24 hours, and days 3 and 7 after reperfusion. Average serum ALT (sALT) levels (**B**) and average Suzuki scores (**C**) in different experimental groups at indicated time points after reperfusion. (**D**) Representative liver histological images (H&E staining; original magnification, ×40; scale bar: 0.2 mm) of different experimental groups at indicated time points after reperfusion. *n* = 6–8 livers/group. *P < 0.05. (**E**) Average ratios of target gene to *HPRT* expression (by a removing PCR) in livers of different experimental groups at days 3 and 7 after reperfusion. Data represent mean ± SEM. Representative results from 4 livers/group. *P < 0.05 (Student’s *t* test).

To determine whether the inflammation resolution was associated with Gsk3β inactivation in liver IRI, we analyzed Gsk3β, N-terminal, S9 inhibitory phosphorylation in IR livers with extended reperfusion time (6 hours to days 3–7 after 90 minutes of ischemia). Western blot result showed that the level of Gsk3β S9 phosphorylation peaked at 6 hours (versus sham) when liver injury was greatest (20) and declined after 12–24 hours. A second peak was detected at days 3–7 after reperfusion when liver IRI started to resolve (Figure 1A). This indicates that Gsk3β inhibitory phosphorylation might be associated with both the activation and resolution of liver IRI.

To test the functional consequences of Gsk3β inactivation in liver IRI, we compared myeloid Gsk3β WT (Gsk3b^fl/fl^) and KO (Lyz-Cre^+^ Gsk3b^fl/fl^) mice at both the activation and resolution stages of the disease. Histological analysis revealed that Gsk3β deficiency resulted in not only significant reduction in liver injuries at 6 hours but also accelerated the restoration of liver homeostasis (Figure 1, B and D). Average Suzuki scores were significantly lower in IR livers of the Gsk3β KO versus WT mice at day 3 after reperfusion. At the molecular level, there were lower levels of TNF-α and higher levels of IL-10 in IR livers of KO mice at this time point (Figure 1E).

Because macrophage efferocytosis is critical for the resolution of inflammatory tissue injury, we measured liver gene expression levels of the efferocytosis receptors MerTK and TIM-4. Indeed, both genes were induced to peak levels in the IR livers of KO mice at day 3 after reperfusion, as compared with 7 days in their WT counterparts (Figure 1E). Additionally, myeloid Gsk3β deficiency diminished the upregulation of profibrotic genes α-SMA and collagen 1A1 (Figure 1E) at day 3 after reperfusion in IR livers. These results indicate that Gsk3β regulates the inflammation resolution in liver IRI.

To differentiate the possibility that the accelerated resolution of liver IRI in the Gsk3β-deficient mice was due to reduced injuries early in the activation stage, we administered a single dose of anti–IL-10 Abs prior to the onset of liver ischemia. This was based on our previous finding that IL-10 was critical for the immune regulatory and cytoprotective effect of the Gsk3β pharmacological inhibitor in liver IRI (20). Indeed, anti–IL-10 exacerbated liver IRI in both the KO and WT mice and diminished their differences at 6 hours after reperfusion (Figure 1B). However, the KO mice still recovered much better than their WT counterparts from liver IRI, as measured by the histological analysis of IR livers at days 3, 5, and 7 after reperfusion. The average Suzuki scores (determined from hematoxylin and eosin [H&E] staining) were significantly lower in the KO group compared with the WT cohort at all these later time points (Figure 1, C and D). The anti–IL-10 treatment resulted in higher TNF-α and lower IL-10 gene expression at day 3 in IR livers, as compared with control Ig-treated livers, in KO and WT mice (Figure 1E). However, the gene-induction kinetics of MerTK and TIM-4 remained accelerated, whereas fibrosis gene expression levels remained lower in KO compared with WT livers at days 3 and 7 after reperfusion (Figure 1E). Thus, Gsk3β regulates the resolution of liver inflammatory response against IR independent of its role in the activation stage of the disease.

### Myeloid Gsk3β regulates iMΦs in the resolution of liver IRI.

**Figure 2:**
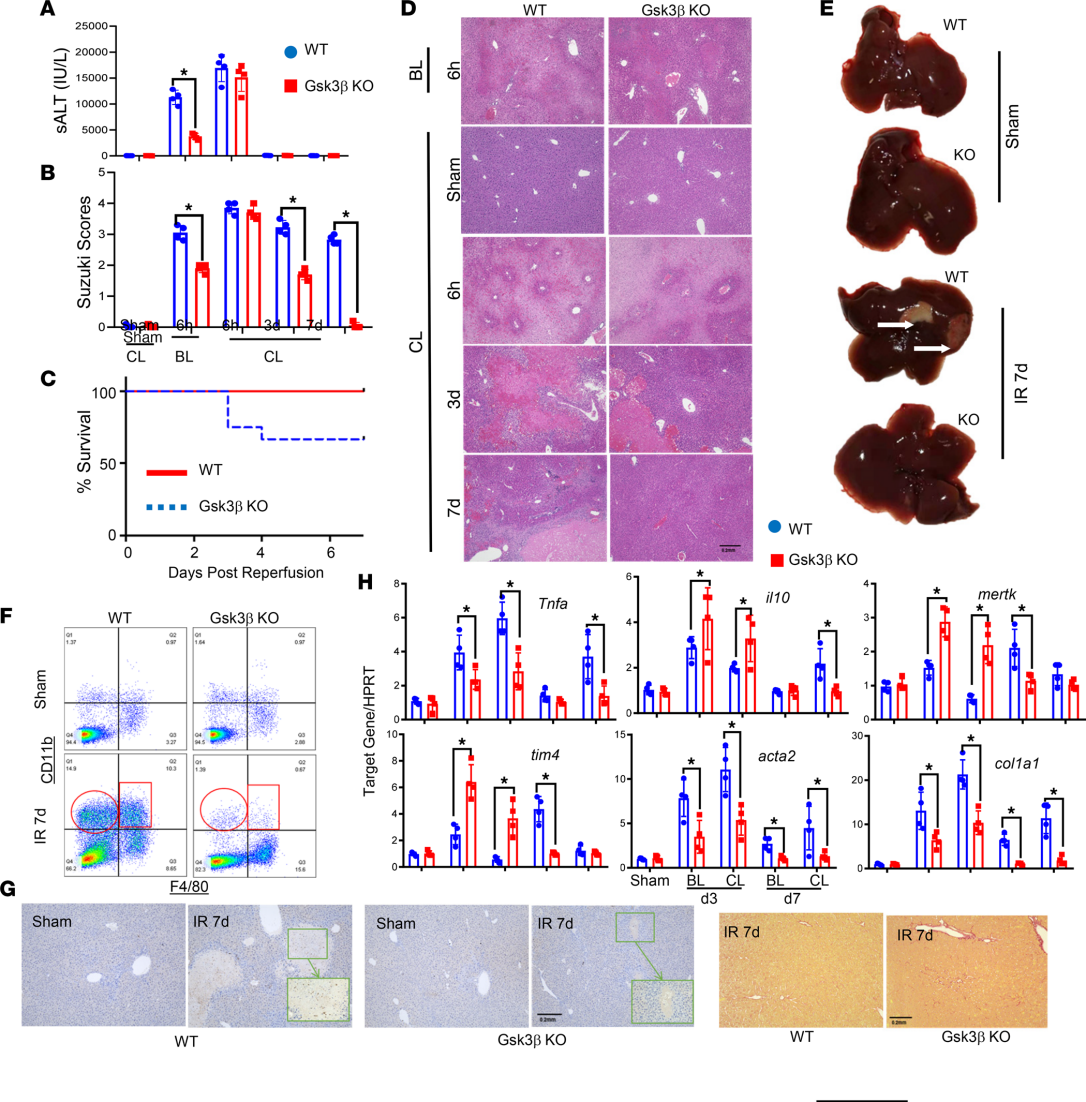
Myeloid Gsk3β regulates the resolution function of iMΦs in liver IRI. Myeloid Gsk3β WT and Gsk3β-KO mice were treated with either blank liposomes or CLs 48 hours before the onset of liver ischemia. IR livers were harvested at 6 hours, 3 days, and 7 days after reperfusion. (**A**) Average levels of serum ALT (sALT). (**B**) Average Suzuki scores of different experiment groups. (**C**) The Kaplan-Meier survival curves of CL-treated WT and KO mice after IR. (**D**) Representative liver histological images (H&E staining; original magnification, ×40; scale bar: 0.2 mm) of different experimental groups at indicated time points after reperfusion. (**E**) Liver gross appearance of sham or IR livers at day 7 after reperfusion of different experimental groups. (**F**) FACS plots of NPCs isolated from sham (48 hours after CL) or IR livers at day 7 after reperfusion of different experimental groups. Myeloid cells were first gated in the FSC/SSC plot and analyzed for F4/80 and CD11b expression. (**G**) Immunohistochemical staining of Ly6G^+^ cells in sham and IR livers at day 7 (left panel), and Sirius red staining of IR livers at day 7 (right panel), after reperfusion of different experimental groups (original magnification, ×40; scale bar: 0.2 mm). *n* = 6–8/group. *P < 0.05. (**H**) Average ratios of target gene to HPRT in sham and IR livers at day 7 after reperfusion of different experimental groups. Data represent mean ± SEM. Representative results from 4 livers/group. *P < 0.05 (Student’s *t* test). BL, blank liposome.

Because both liver-resident KCs and iMΦs are involved in the pathophysiology of liver IRI, we tested whether Gsk3β differentially regulated these 2 macrophage subsets in the activation and resolution stages of the disease process. KCs were depleted by clodronate liposomes (CLs) 48 hours prior to the onset of liver ischemia. Liver IRI was measured at 6 hours after reperfusion. Consistent with published results (24, 25), including our own (26), the CL treatment increased liver IRI in WT mice. Myeloid Gsk3β–deficient mice also had more severe liver damage after the CL treatment. Importantly, the differences between the WT and KO mice in liver IRI was diminished at this time point (6 hours) after reperfusion, with similar average levels of serum alanine aminotransferase (ALT) and liver histopathological grading (Figure 2A). However, the resolution of liver IRI remained drastically different (Figure 2, B–D). In fact, the mortality rate of KC-depleted WT mice was approximately 25%–30% at days 2–3 after reperfusion, whereas all myeloid Gsk3 KO counterparts survived (Figure 2C). The recovery of liver IRI was accelerated in KO mice. Average Suzuki scores were significantly lower in KO versus WT cohorts at days 3 and 7 after reperfusion (Figure 2, B, D, and E). Hepatocellular damage was fully repaired in KO mice at day 7, as shown by H&E staining and gross appearance (Figure 2, D and E), whereas in the WT counterparts, full repair was not complete until day 14 (27).

The resolution of liver inflammation was also measured at the cellular level. Liver NPCs were isolated on day 7 after reperfusion and analyzed by FACS. On the basis of F4/80 and CD11b staining, it was clear that KCs were depleted by the CL treatment in the livers (sham;48 hours after injection). Although there were significant amounts of iMΦs (CD11b^+^F4/80^+^) and neutrophils (CD11b^+^F4/80^–^) detected in the IR livers of WT mice, these infiltrated cells were nearly absent in livers of KO mice at day 7 after reperfusion (Figure 2F). This indicates accelerated reprogramming of iMΦs and clearance of neutrophils by myeloid Gsk3β deficiency. The immunohistochemical staining of Ly6G^+^ cells also showed significantly fewer neutrophils in the IR livers of CL-treated KO versus WT mice (Figure 2G).

At the molecular level, the CL treatment resulted in increases in proinflammatory changes and fibrosis and decreases in proresolving gene expression in IR livers of both strains of mice at day 3 after reperfusion (versus blank liposome–treated livers; Figure 2H). However, the relative levels of TNF-α, α-SMA, and Col1A1 remained lower, whereas levels of IL-10, MerTK, and TIM-4 were higher, in the livers of KO versus WT mice (Figure 2H). Sirius red staining confirmed that fibrosis, indeed, was reduced in myeloid Gsk3β–deficient IR livers at day 7 after reperfusion (Figure 2G).

Because Gsk3β may potentially regulate macrophage proliferation, we quantitated F4/80^+^ cells in IR livers of CL-treated mice by immunofluorescence staining. Again, F4/80^+^ cells were depleted by CLs and detected in the IR livers of the 2 strains of mice at day 7 after reperfusion, without significant differences in the numbers of positive cells (Supplemental Figure 1; supplemental material available online with this article; https://doi.org/10.1172/jci.insight.151819DS1). These results indicate that myeloid Gsk3β regulates iMΦs in the resolution of liver IRI. Gsk3β deficiency upregulates the expression of immune-regulatory and proresolving genes and facilitates the reparative reprogramming and functions in iMΦs.

### Gsk3β regulates BMMs in the resolution of liver IRI.

Liver iMΦs originate from BM. To functionally specify whether Gsk3β regulated BMMs in the resolution of liver IRI, we reconstituted CD11b-DTR mice with either Gsk3β-WT or -deficient BMMs. The recipient mice were conditioned with diphtherial toxin 24 hours prior to the onset of liver ischemia, and BMMs were injected after 24 hours of reperfusion. IR livers were harvested on days 3 and 7 after reperfusion and subjected to histological and molecular analyses.

**Figure 3:**
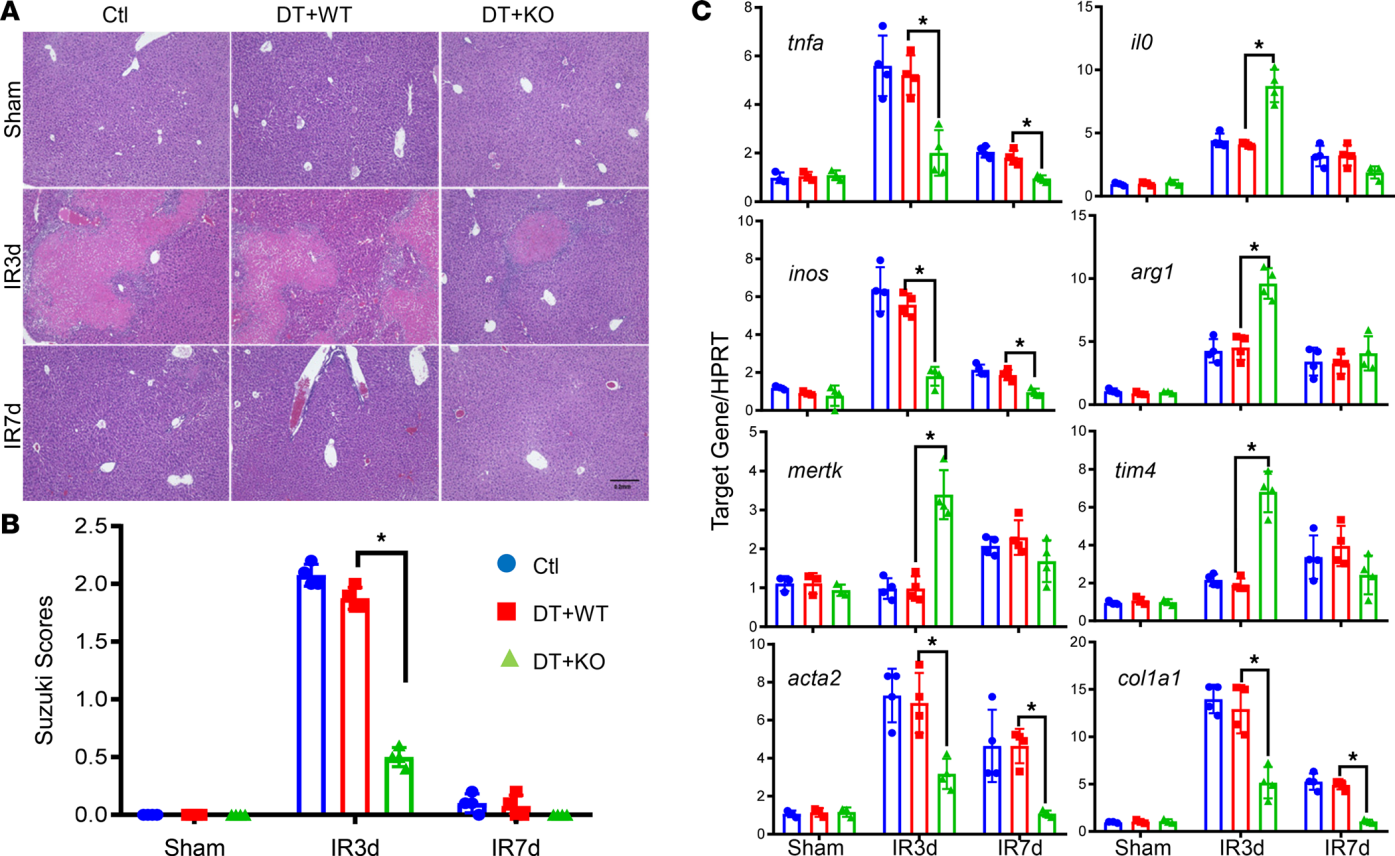
Gsk3β regulates BMMs in the resolution of liver IRI. CD11b-DTR mice were treated with diphtheria toxin at 24 hours prior to the onset of liver ischemia. BMMs derived either from myeloid Gsk3β WT orGsk3β-KO mice were injected at 24 hours after reperfusion, as described in Methods. Sham and IR livers harvested at days 3 and 7 after reperfusion were analyzed. (**A**) Liver histological images (H&E staining; original magnification, ×40; scale bar: 0.2 mm). (**B**) Average Suzuki scores of different experiment groups at indicated time points after reperfusion. *n* = 6×8/group. *P < 0.05. (**C**) Average ratio of target gene to HPRT in sham and IR livers at days 3 and 7 after reperfusion of different experimental groups. Data represent mean ± SEM. Representative results from 4 livers/group. *P < 0.05 (Student’s *t* test). Ctl, control; DT, diphtheria toxin.

Indeed, KO BMMs were much better in helping IR livers recover from IRI, and average Suzuki scores were significantly lower at day 3 in the KO versus WT BMM-reconstituted mice (Figure 3, A and B). Liver IRI was resolved at day 7 after reperfusion in both cohorts. This was accompanied by lower proinflammatory and profibrosis gene expression (TNF-α, iNOS, α-SMA, Col1A1) and higher immune-regulatory and proresolving (IL-10, Arg1, MerTK, TIM-4) gene expression in Gsk3β-deficient compared with WT-cell reconstituted livers (Figure 3C). These results confirm that Gsk3β regulates the proresolution functions of BMMs in liver IRI.

### Myeloid Gsk3β deficiency facilitates the development of proresolving functions in BMMs in liver IRI.

**Figure 4:**
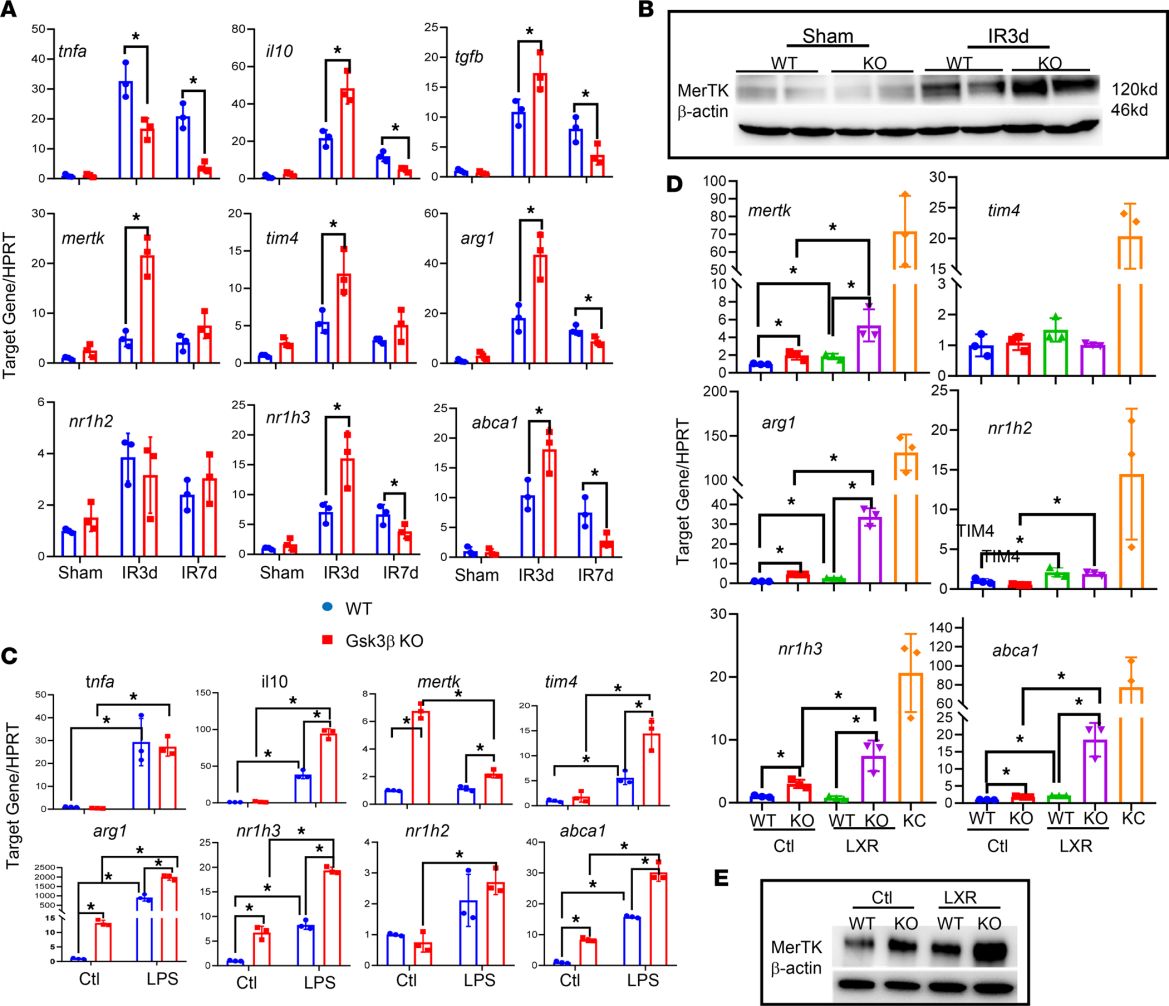
Myeloid Gsk3β deficiency enhances proresolving gene expressions in macrophages. NPCs were isolated from sham and IR livers at days 3 and 7 after reperfusion in CL-treated WT or myeloid Gsk3β-KO mice, as described in Methods. (**A**) Average ratios of target gene to HPRT. (**B**) Western blot of MerTK levels in liver NPCs of different experimental groups at indicated time points after reperfusion. (**C** and **D**) BMMs derived from WT or myeloid Gsk3β-KO mice were stimulated in vitro for 24 hours with LPS (**C**) or the LXR agonist DMHCA (**D**). Average ratios of target gene to HPRT of different experiment groups are plotted. KCs isolated from WT sham livers were used as the control in **D**. (**E**) Western blot of MerTK in LXR-stimulated BMMs. Gene expression was measured by quantitative reverse-transcription PCR. Data represent mean ± SEM. Representative results from 3–4 livers/group. *P < 0.05 (Student’s *t* test). Ctl, control.

At the cellular level, we isolated liver NPCs from CL-treated myeloid Gsk3β–deficient and WT mice at days 3 and 7 after reperfusion and compared gene expression profiles by quantitative reverse-transcription PCR. Liver NPCs from CL-treated sham livers were used as the baseline (non-macrophages) control. As shown in Figure 4, myeloid Gsk3β deficiency downregulated TNF-α at both days 3 and 7 but upregulated IL-10, TGF-β, Arg-1, MerTK, and TIM-4 expression at day 3 after reperfusion in liver NPCs (Figure 4A). Western blot analysis of day 3 samples confirmed the elevated level of MerTK expression in the KO cells compared with WT cells (Figure 4B). Additionally, we measured the expression and activation of LXR, a key transcription factor for the differentiation of BMMs to KCs (28, 29) and the proresolution gene program in macrophages (e.g., efferocytosis) (30, 31). Although LXRβ (Nr1h2) is constitutively expressed, LXR-α (Nr1h3) is inducible in response to various stimuli. Indeed, Nr1h3 was induced in liver NPCs, and NrlH2 much less so, during the resolution stage of IRI, and Gsk3β deficiency enhanced the induction rate at day 3 after reperfusion (Figure 4A). This was accompanied by higher induction of LXR-targeted gene expression of Abca1 and MerTK in the KO cells compared with WT cells. It is worth pointing out that the expression of these immune-regulatory and proresolving genes was similar or lower in the KO than WT cells at day 7, which might correlate with the different kinetics of the resolution of liver IRI in these 2 strains of mice.

To confirm MerTK induction in iMΦs and its functional significance, we switched to the LPS-induced peritonitis model, in which peritoneal macrophages could be extracted without collagenase digestion (which may affect cell surface MerTK expression) (32–34). The peritoneal resident macrophages and iMΦs can be distinguished by TIM-4 expression (35). FACS analysis revealed clearly that Gsk3β-deficient iMΦs (TIM-4^–^F4/80^+^) were induced to express MerTK at substantially higher levels than in the WT counterparts at the resolution stage of the disease (48 hours after LPS injection; Supplemental Figure 2). Their efferocytosis functions, measured in vitro, also were higher than in WT cells.

Because TLR4 is critical for iMΦ activation in liver IRI, and iMΦ reprogramming depends on liver-specific stimuli (28, 29), we studied BMMs in vitro in response to LPS or the LXR agonist *N*,*N*-dimethyl-3β-hydroxy-cholenamide (DMHCA). The induction of an immunoregulatory or proresolving gene program was measured at 24 hours after stimulation. KCs isolated from WT sham livers were included to represent liver homeostatic macrophages. Interestingly, Gsk3β-deficient BMMs expressed constitutively higher levels of Arg-1, Nr1h3, MerTK, and Abca1, indicating their differentiation bias toward an immune regulatory or reparative phenotype during BM cell culture with macrophage CSF (Figure 4, C and D). The proinflammatoryTLR4 stimulation increased expression of Arg-1, Nr1h3, and Abca1 but decreased MerTK expression in both types of cells. The relative levels of these transcripts remained significantly higher in the LPS-stimulated KO cells than in WT cells (Figure 4C). TNF-α expression was similar in the 2 types of cells at this time point. However, IL-10 and TIM-4 were expressed at significantly higher levels in the KO cells. The proresolution LXR stimulation, on the other hand, induced MerTK, but not TIM-4, expression in BMMs. Gsk3β-deficient cells had significantly higher levels of MerTK than the WT counterparts both before and after LXR stimulation (Figure 4C), which was confirmed by Western blot analysis (Figure 4E). Levels of Arg-1, Nr1h3, and Abca1 were also higher in LXR-stimulated Gsk3β KO than in WT BMMs (Figure 4D). Overall, Gsk3β-deficient BMMs were more similar to KCs than to the WT counterpart in the LXR-induced gene expression profile. Thus, Gsk3β deficiency facilitates the induction of immune regulatory and reparative genes in BMMs both constitutively and upon proinflammatory or proresolving stimulation.

**Figure 5:**
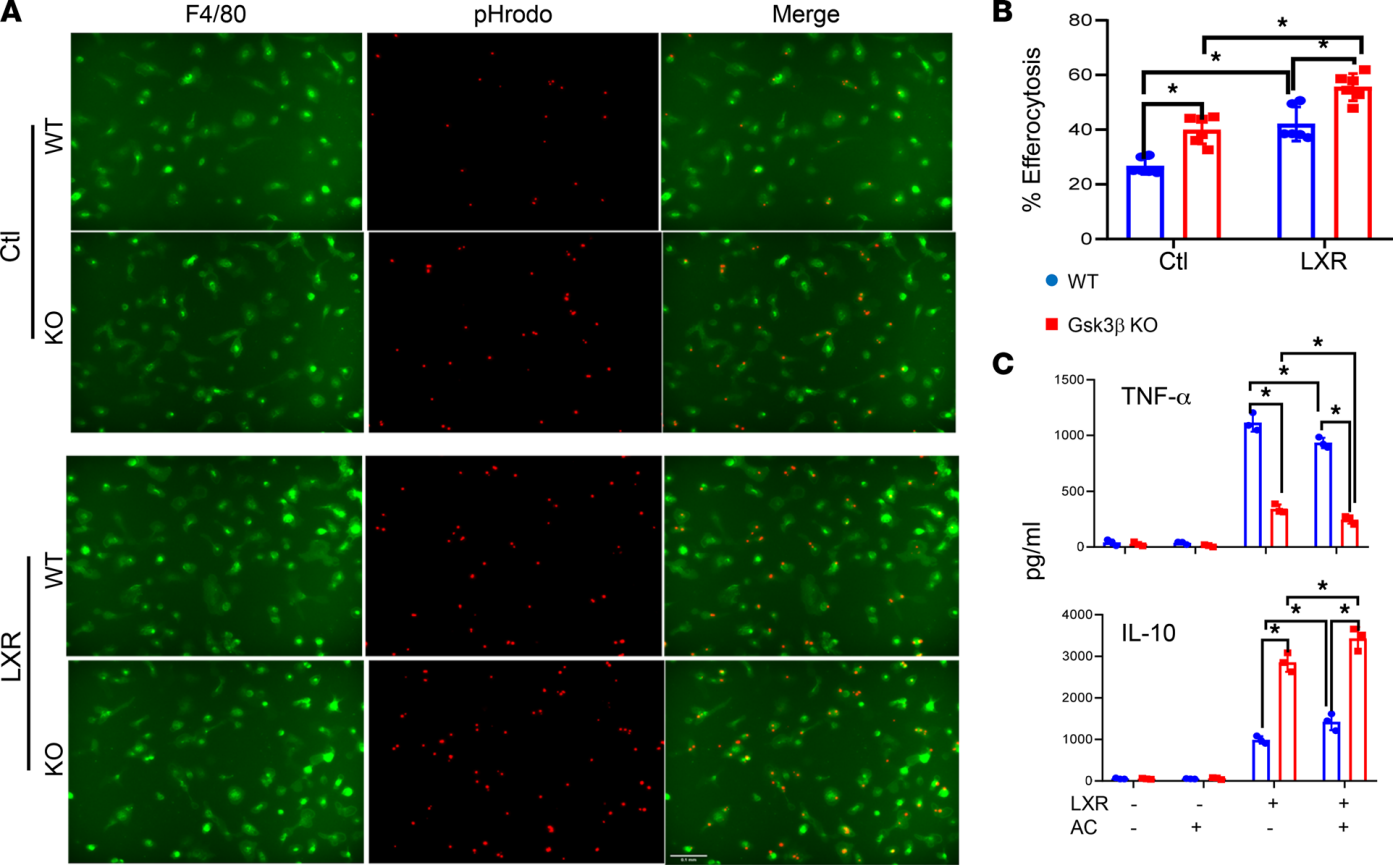
Myeloid Gsk3β deficiency enhances proresolution functions in BMMs in vitro. BMMs derived from WT or myeloid Gsk3β-KO mice were stimulated in vitro for 24 hours with a control (Ctl) or the LXR agonist DMHCA and tested in an in vitro efferocytosis assay by incubating with pHrodo-labeled apoptotic thymocytes, as described in Methods. Cells were stained with FITC-labeled anti–F4/80, and efferocytosis was quantitated under a confocal microscope. (**A**) Representative fluorescence images of F4/80-stained BMMs. (**B**) Average percentage of efferocytes in total macrophage population. (**C**) Average TNF-α and IL-10 levels in the culture supernatants of WT and Gsk3β KO BMMs stimulated with LPS for 24 hours in the absence or presence of apoptotic thymocytes. Cytokine levels were quantitated by ELISA. Data represent mean ± SEM. Representative results from 4 livers/group. *P < 0.05 (Student’s *t* test). AC, apoptotic cells.

The proresolution function of BMMs before and after LXR stimulation was measured in an in vitro efferocytosis assay. Confocal fluorescence microscopy indicated that Gsk3β-deficient cells had higher efferocytosis capacities both constitutively and after LXR stimulation than their WT counterparts. The LXR stimulation promoted efferocytosis in both types of cells, consistent with its ability to induce MerTK expression (Figure 5, A and B). The immune regulatory effect of efferocytosis was analyzed by co-incubating BMMs with apoptotic thymocytes during LPS stimulation. ELISA results showed that Gsk3β-deficient BMMs produced higher levels of IL-10 and lower levels of TNF-α than did their WT counterparts, and the presence of apoptotic cells further increased IL-10 and decreased TNF-α production by both types BMMs (Figure 5C). Thus, Gsk3β deficiency enhances proresolution functions of BMMs both constitutively and upon stimulation.

### Pharmacological inhibition of Gsk3 facilitates the resolution of liver IRI in an MerTK-dependent manner.

**Figure 6:**
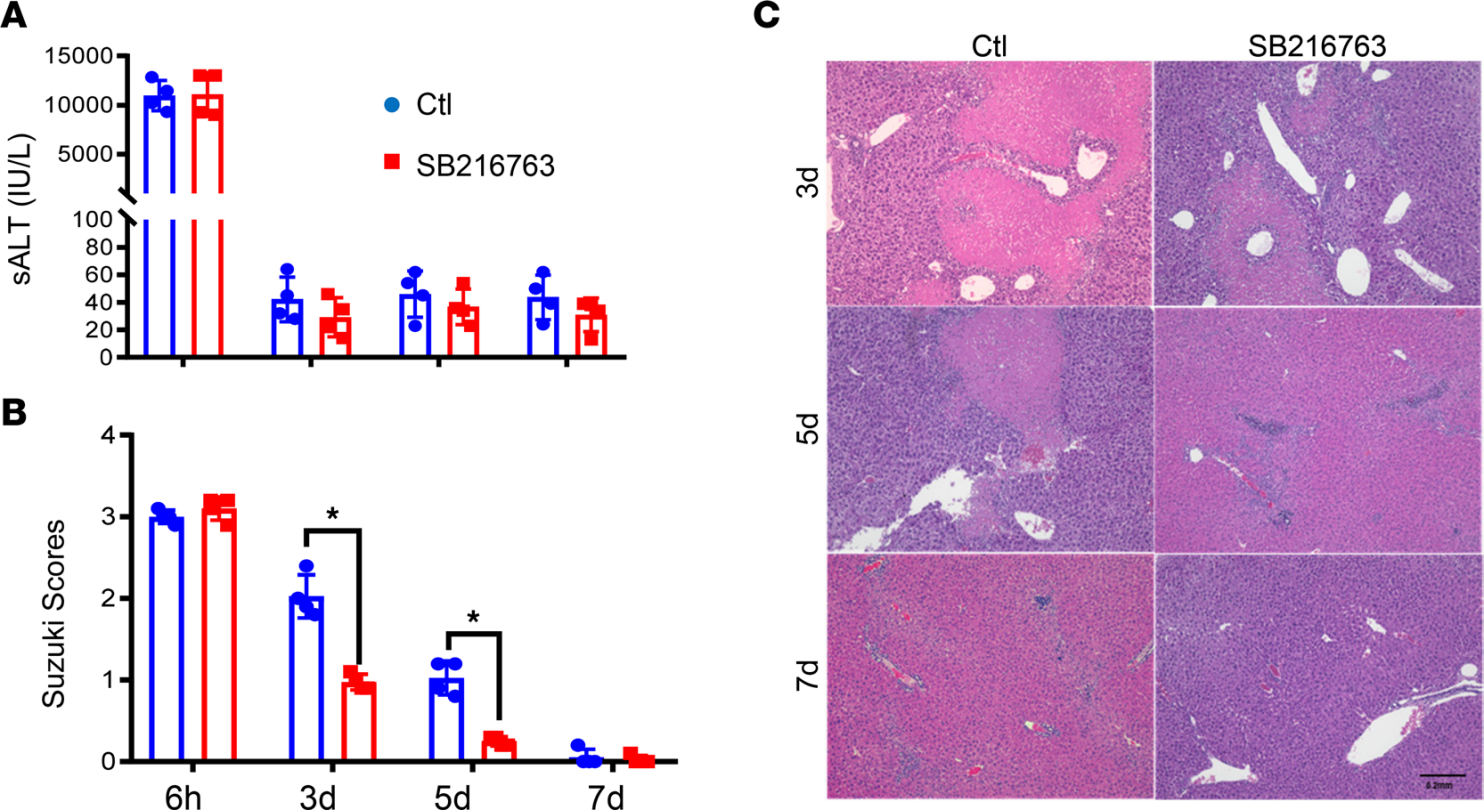
Pharmacological inhibition of Gsk3 facilitates the resolution of liver IRI. WT B6 mice were subjected to 90 minutes of liver ischemia, as described in Methods. At 24 hours after reperfusion, they were divided into 2 groups and treated with vehicle control (Ctl) or SB216763. IR livers were harvested at days 3, 5, and 7 after reperfusion and analyzed by histological evaluation. Average serum ALT (sALT) levels (**A**) and average Suzuki scores (**B**) of the 2 experimental groups at indicated time points after reperfusion. (**C**) Representative liver histological images (H&E staining) of different experiments at indicated time points after reperfusion. Data represent mean ± SEM. Representative results from 6–8 livers/group. *P < 0.05 (Student’s *t* test).

To test whether pharmacological inhibition of Gsk3 had therapeutic effects on the resolution of liver IRI, we treated WT B6 mice with 3 consecutive doses of SB216763 (25 μg/g i.p.) starting at 24 hours after reperfusion (after liver IRI already peaked). Serum ALT levels and liver histopathology were analyzed at 6 hours and on days 3, 5, and 7 after reperfusion (Figure 6). With similar levels of liver IRI at 6 hours (according to serum ALT level), Gsk3 inhibition facilitated the recovery of liver IRI. Compared with vehicle controls, repair of hepatocellular damage was improved in SB216763-treated livers, with lower average Suzuki scores at each of these time points (Figure 6). These results extend the therapeutic efficacy of pharmacological Gsk3 inhibition in the resolution of liver IRI.

**Figure 7:**
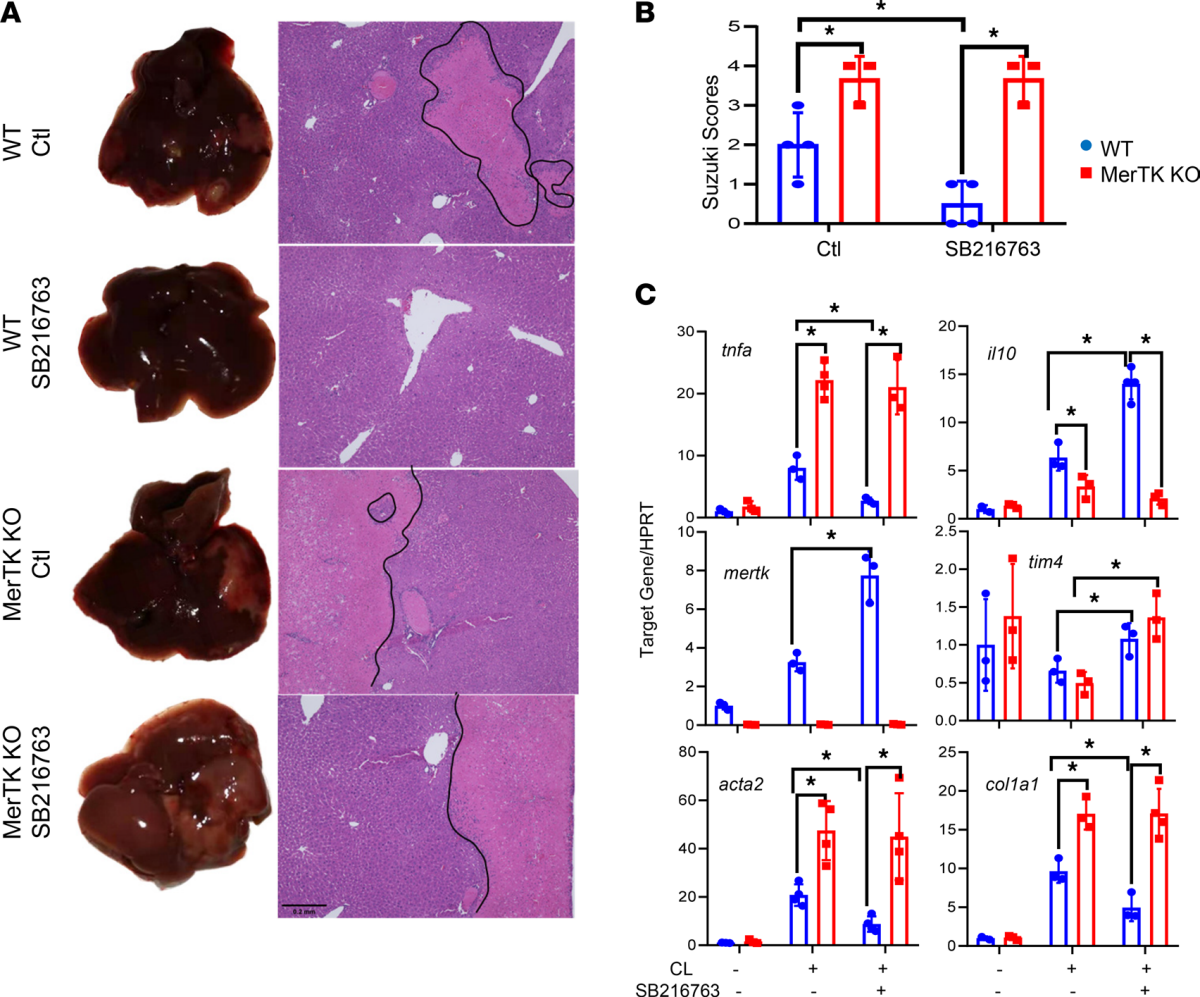
MerTK is critical for the proresolving therapeutic effect of pharmacological Gsk3 inhibition. WT and myeloid MerTK-KO mice were treated with CLs 48 hours prior to the onset of liver ischemia. Vehicle control (Ctl) and SB216763 were administered at 24 hours and on days 3 and 5 after reperfusion, as described Methods. IR livers were harvested on day 7 after reperfusion. (**A**) Liver gross appearance and histological images of different experiment groups. (**B**) Average Suzuki scores of IR livers of different experimental groups at day 7 after reperfusion. (**C**) Average ratios of target gene to HPRT in IR livers of different experimental groups at day 7 after reperfusion. Liver gene expression was determined by quantitative reverse-transcription PCR. Data represent mean ± SEM. Representative results from 4–6 livers/group. *P < 0.05 (Student’s *t* test).

To explore the molecular mechanism of Gsk3β inactivation in promoting macrophage proresolution functions, we compared the therapeutic effect of the Gsk3 inhibitor between myeloid MerTK KO and WT mice. To focus on iMΦs, we depleted KCs by CLs prior to the onset of liver ischemia, followed by administration of 3 doses of SB216763 (25 mg/kg i.p.) starting at 24 hours after reperfusion. Liver histopathology and inflammatory response were analyzed at day 7 after reperfusion. Unlike KC-intact cohorts, Gsk3 inhibition facilitated liver recovery from IRI only in KC-depleted WT mice, not in MerTK-deficient mice, as measured by average Suzuki scores at day 7 after reperfusion (Figure 7A). Myeloid MerTK-KO mice had significantly more liver IRI than did their WT counterparts, regardless of the treatment, and the significant difference in the restoration of liver homeostasis between SB216763- and vehicle-treated IR livers detected in CL-treated WT mice at day 7 was diminished by myeloid MerTK deficiency (Figure 7B). At the molecular level, Gsk3 inhibition increased MerTK, TIM-4, and IL-10 expression but decreased TNF-α, α-SMA, and Col1A1 gene expression in IR livers of WT, but not MerTK KO (except TIM-4), mice (Figure 7C). These results indicate that the induction of efferocytosis receptor MerTK is critical for the proresolving mechanism of Gsk3 inactivation in liver iMΦs.

## Discussion

Our study describes a murine model in which Gsk3β regulates the inflammation resolution, independent of Gsk3β’s role in the activation stage, in liver IRI by controlling the development of proresolving functions in iMΦs. This is illustrated biochemically by the detection of second peak of Gsk3β inhibitory phosphorylation in the resolution stage, as well as functionally by the clinical impact of Gsk3β inactivation in liver IRI with both cell type–specific genetic deficiency in mice and chemical inhibition. Our data further show that the efferocytosis receptor MerTK is one of the key downstream mediators of this proresolving mechanism of Gsk3 inactivation, because Gsk3β regulates MerTK induction in BMMs, and myeloid-specific deficiency of MerTK diminishes the therapeutic benefits of Gsk3 inhibition in the resolution of liver IRI.

Roles of macrophages in liver tissue repair have been documented in several models. A seminal work using CD11b-DTR mice showed that depletion of CD11b^+^ cells (iMΦs) during the induction phase of CCl_4_-induced liver fibrosis attenuated the disease severity, whereas cell depletion at the recovery phase delayed tissue repair (36), suggesting that liver iMΦs could play distinctive roles at different stages of the disease process. iMΦs and KCs were involved in the clearance of apoptotic cells and neutrophils and repair of hepatocellular damages in the acetaminophen- or heat-induced acute liver injury model (37–40). CCR2^+^ monocytes were shown to infiltrate inflamed livers and differentiate into reparative CX3CR1^+^ macrophages. KCs were also shown to be the dominant reparative cells, by expressing MerTK (38). In liver IRI models, neuroimmune guidance cue ntrin-1 regulated inflammation resolution and regeneration by controlling the infiltration of Ly6C^low^ macrophages, which had higher capacity of efferocytosis (versus Ly6C^high^ cells) in vitro (41). Our study is among the first to dissect roles of Gsk3β in liver iMΦs in the resolution of IRI using KC-depleted myeloid specific gene KO models.

Although CLs effectively depleted KCs, they also reduced iMΦs early after reperfusion (26). However, we found abundant iMΦs in IR livers at the resolution stage (i.e., at day 7 after reperfusion) (Figure 2F), and myeloid Gsk3β deficiency facilitated liver recovery from IRI in these KC-depleted mice. The reconstitution of CD11b^–^DTR mice with BMMs provides direct evidence of the specific role of Gsk3β in iMΦs in the resolution of liver IRI. It is important to point out that these data do not exclude the role of KCs in the late stage of liver IRI, which may also be subjected to Gsk3β regulation. In fact, the CL treatment resulted in increases of hepatocellular damage and delay of the inflammation resolution in our model (Figure 2). As KC depletion abrogates the differences between myeloid Gsk3β WT and Gsk3β-KO mice in liver IRI at 6 hours after reperfusion, it implicates the role of Gsk3β in KCs in the activation stage of the disease. Whether Gsk3β specifically regulates KC proresolution function remains to be determined.

The proresolving iMΦs can be either descendant of proinflammatory precursors infiltrated earlier or of new infiltrates during the resolution of liver IRI (42, 43). Although our data do not differentiate these 2 possibilities, we do show that myeloid Gsk3β deficiency enhances immunoregulatory and proresolution properties of iMΦs in vivo in IR livers, both functionally and genetically (as seen in gene expression profiles). Our in vitro experiments further reveal that Gsk3β regulates the differentiation of BMMs toward the proresolution type in response to both inflammatory (TLR4) and liver-specific (LXR) stimuli. Interestingly, the expression of LXR-α, which is critical for macrophage efferocytosis function (44, 45) and KC differentiation (28), was significantly increased by Gsk3β deficiency in liver iMΦs (Figure 4A) in vivo and in BMMs in vitro upon TLR4 or LXR stimulation. The activation of LXR was demonstrated by the upregulation of LXR-targeted gene expression, including of Abca1 and MerTK. Functionally, we demonstrate that Gsk3β deficiency increases BMM efferocytosis both before and after LXR stimulation and enhances the immunoregulatory response of BMMs upon TLR4 stimulation in the presence or absence of apoptotic cells. It remains to be determined how Gsk3β regulates LXR-α expression. One potential pathway is via Notch 1 signaling, which facilitates LXR-α expression in liver iMΦs (28), and Gsk3β inhibition enhances Notch 1 activation by mobilizing the receptor from endosomal stores (46). Interestingly, liver IRI is aggravated in myeloid Notch-deficient mice (47).

Roles of MerTK in liver injury models seem to be context specific. It is critically involved in liver recovery from inflammatory tissue injuries in LPS/D-Gal and anti–Fas Ab–induced models (48). However, the same global MerTK deficiency did not significantly affect the resolution of acetaminophen-induced liver injuries (38, 48). Growth arrest–specific gene 6 (GAS6), a MerTK ligand, plays an important role in the tissue repair of acute CCl_4_-triggered liver injuries (49) and protects livers from IRI by diminishing proinflammatory cytokine productions by macrophages in response to LPS (50). We found in the present study that the resolution of liver IRI was significantly delayed in myeloid MerTK–deficient mice. Importantly, pharmacological inhibition of Gsk3, which facilitated liver recovery from IRI in WT mice, did not exert the proresolving effect in these MerTK-KO mice. Thus, MerTK induction constitutes one of the key proresolution mechanisms of Gsk3β inactivation in liver iMΦs. It is important to clarify that Gsk3β deficiency only accelerates and enhances MerTK induction and expression. Gsk3β is naturally inhibited by phosphorylation upon stimulation in WT cells, and our preliminary experiments have shown that Gsk3β-deficient KCs and peritoneal macrophages were similar to their WT counterparts, which are all MerTK^hi^, in their capacity of efferocytosis (data not shown).

**Figure 8:**
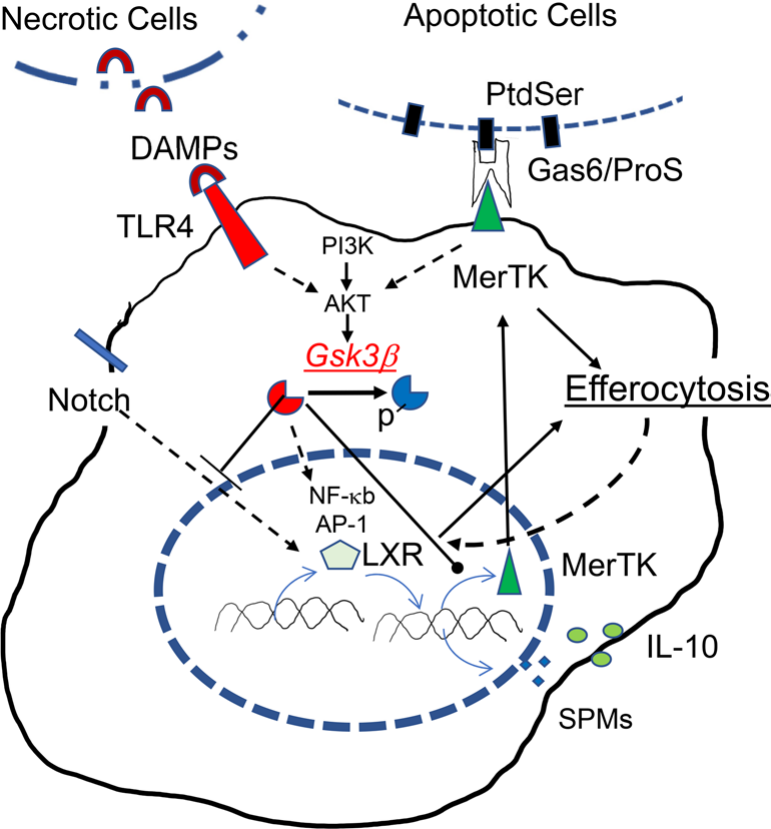
Gsk3β as the converging point in the differentiation and the execution of pre-resolving functions in macrophages. Gsk3β is able to regulate (1) TLR-mediated sterile inflammation (DAMPs from necrotic cells); (2) Notch mediated signaling (to upregulate LXR); as well as (3) MerTK-mediated resolution functions (binds PtdSer on apoptotic cells via Gas6/ProS) and LXR-mediated resolution functions, downstream or independent of PI3K/Akt, are potentially regulated by Gsk3β. Gsk3β inactivation downregulates the proinflammatory, but upregulate the immunoregulatory or reparative, gene programs, including IL-10, MerTK, and LXR-α, and possibly specific proresolving mediators (SPMs).

Obviously, there are many other potential proresolution mediators downstream of Gsk3β. In addition to the obvious candidates, such as IL-10 and TGF-β, LXR activation promotes the resolution of acute sterile and lung inflammation (51, 52), and pharmacological activation of LXR attenuates inflammatory disorders in multiple organ (53). Interestingly, LXR can be induced by MerTK activation and efferocytosis in macrophages in a PI3 kinase-Akt–dependent manner (52), and LXR is important for efferocytosis-initiated reprogramming of macrophages to the reparative type (31, 54, 55). We hypothesize that Gsk3β may act as the potential converging point in the differentiation and the execution of pre-resolving functions in macrophages (Figure 8). Gsk3β, as the key kinase downstream of the PI3K/Akt signaling pathway, regulates LXR expression and activation and MerTK induction upon proinflammatory and differentiation stimulation (9, 18, 19). The same pathway is also critically important for the execution of MerTK-initiated proresolving functions (namely, efferocytosis and synthesis of immune regulatory and proresolving mediators) (56, 57).

In summary, our data reveal in a mouse model a proresolution mechanism of Gsk3β inactivation in liver IRI by regulating the induction of an immune regulatory and tissue reparative gene program in BM-derived iMΦs. This knowledge not only advances our understanding of the pathophysiology of liver IRI, it also adds a new dimension in the therapeutic exploration of Gsk3β inhibition in tissue inflammatory diseases.

## Methods

### Animals.

Male C57BL/6J (WT), CD11b-DTR, and Lyz-Cre mice (6–8 weeks old) were purchased from the Jackson Laboratory. Myeloid Gsk3β or MerTK-KO mice were created by crossing floxed Gsk3β (from Jim Woodgett, University of Toronto, Samuel Lunenfeld Research Institute, Toronto, Ontario, Canada) or floxed-MerTK (from Carla V. Rothlin, Yale University, New Haven, Connecticut, USA) mice with Lyz-Cre mice. All mice were housed in the UCLA animal facility under specific pathogen-free conditions and received humane care according to the criteria outlined in the *Guide for the Care and Use of Laboratory Animals* (National Academies Press, 2011).

### Mouse liver partial warm-ischemia model.

After a midline laparotomy, mice were injected with heparin (100 μg/kg) and an atraumatic clip was used to interrupt arterial and portal venous blood supply to the cephalad liver lobes. After 90 minutes of ischemia, the clip was removed to initiate hepatic reperfusion. Sham controls underwent the same procedure but without vascular occlusion. Mice were sacrificed after 6 hours to 7 days of reperfusion, and liver and serum samples were collected. Serum ALT levels were measured with an autoanalyzer by ANTECH Diagnostics. Portions of liver specimens were fixed in 10% buffered formalin and embedded in paraffin. Liver sections (4 μm) were stained with H&E. The severity of liver IRI was graded blindly using the Suzuki criteria on a scale from 0 to 4. No necrosis, congestion, or centrilobular ballooning is given a score of 0, whereas severe congestion and >60% lobular necrosis is given a score of 4. Anti–IL-10 Abs (0.5mg/mouse; clone JES5-2A5, Bio-Express) were administered i.p. 1 hour prior to inducing the liver ischemia. Gsk3 inhibitor SB216763 (25 μg/g; Sigma) was administered i.p. at 24 hours, 3 days, and 5 days after reperfusion.

### Liver NPC/KC isolation.

Liver NPCs and KCs were isolated from normal or IR livers of B6 mice by in situ collagenase perfusion. In brief, livers were perfused via the portal vein with calcium- and magnesium-free HBSS supplemented with 2% heat-inactivated FBS, followed by 0.27% collagenase IV (Sigma). Perfused livers were dissected and teased through 70 μm nylon-mesh cell strainers (BD Biosciences). NPCs were separated from hepatocytes by centrifuging at 50*g* for 2 minutes 3 times. NPCs were stained with fluorescence-labeled Abs and analyzed by FACS. To enrich KCs, NPCs were suspended in HBSS and layered onto a 2-layer 25%–50% Percoll gradient (Sigma-Aldrich) in a 50 mL conical centrifuge tube and centrifuged at 1800*g* at 4^°^C for 15 minutes. KCs in the middle layer were collected and allowed to attach to cell culture plates in supplemented DMEM with 10% FBS for 15 minutes at 37^°^C. Nonadherent cells were removed by replacing the culture medium. The purity of KCs in the adherent cells was determined by immunofluorescence staining with anti–F4/80. Most (80%–90%) adherent cells were F4/80 positive.

### BMM cell cultures.

BM cells were isolated from mouse femurs and tibias. Cells were cultured in DMEM supplemented with 10% FBS and 20% L929-conditioned medium for 7 days. BMMs were stimulated with either LPS (200 μg/mL) in the absence or presence of apoptotic thymocytes, or DMHCA(a selective LXR agonist; 1 μM), for 24 hours. Culture supernatants and cells were harvested for additional analysis.

### Macrophage depletion and reconstitution.

Macrophage Depletion Kit (Encapsula NanoSciences) was used to deplete KCs, according to manufacturer’s protocols. In brief, 200 μL/mouse clodronate-encapsulated liposomes or control liposomes were injected i.v. at 48 hours prior to the onset of liver ischemia. In CD11b-DTR mice, 10 μg/g diphtheria toxin was injected i.v. at 24 hours and 0 hours before the onset of liver ischemia; 2.5 × 10^6^ BMMs were injected i.v. at 24 hours after reperfusion. The depletion specificity was documented in the FACS analysis of liver NPCs at day 3 after reperfusion (Supplemental Figure 3).

### Flow cytometry.

Liver NPCs were isolated from sham or IR livers, as described above. We incubated 1×10^6^ cells first with rat anti–mouse CD16/32 for 10 minutes, followed by staining with rat anti–mouse F4/80 (clone BM8), CD11b (clone M1/70), Gr1 (clone RB6-8C5), TIM-4 (clone RMT4-54), MerTK (clone DS5MMER), or isotype-matched control Ab (eBioscience) for 20 minutes. Cells were washed with PBS and subjected to ﬂow cytometry analysis with BD LSR Fortessa (BD Biosciences). A representative FACS plot of liver NPCs at day 3 after reperfusion is shown in Supplemental Figure 4. Clearly, KCs expressed higher levels of MerTK than iMΦs.

### Efferocytosis assay.

Thymocytes (10 /mL) were incubated with dexamethasone (0.1 μM; Sigma-Aldrich) for 16 hours in supplemented DMEM medium plus 10% FBS to induce apoptosis. Apoptotic cells were labeled with 20 ng/mL pHrodo red succinimidyl ester (Thermo Fisher Scientific) for 30 minutes and washed twice with PBS. BMMs (1.5 × 10^5^) were plated on 8-well Permanox Plastic chamber slides (Nunc) and co-cultured with 2 × 10^5^ labeled apoptotic thymocytes for 2 hours in 200 μL of medium. Nonadherent cells were washed off with PBS. BMMs were further incubated with rat anti–mouse CD16/32 for 10 minutes and stained with rat anti–mouse F4/80-FITC or isotype-matched control Ab (eBioscience). After 20 minutes of incubation in the dark, the cells were washed with PBS and fixed for 15 minutes with 4% paraformaldehyde containing 5% sucrose. BMMs and efferocytes were visualized under a confocal fluorescent microscope (KEYENCE). The number of pHrodoSE^+^ BMMs were enumerated; the percentage of efferocytes was calculated as the number of pHrodoSE^+^ BMMs divided by the total number of BMMs.

### Quantitative reverse-transcription PCR.

Total RNA (2.0 μg) was reverse transcribed into cDNA using SuperScript III First-Strand Synthesis System (Invitrogen, Carlsbad, CA). Quantitative PCR was performed using the DNA Engine with Chromo 4 Detector (MJ Research). To a final reaction volume of 20 μL, the following were added: 1× SuperMix (Platinum SYBR Green qPCR Kit, Invitrogen), cDNA, and 0.5 mM of each primer. Amplification conditions were as follows: 50^°^C (2 minutes), then 95^°^C (5 minutes), followed by 50 cycles at 95^°^C (15 seconds), then at 60^°^C (30 seconds). The primers for mouse gene fragments, including *tnfa*, *il10*, *tgfb*, *mertk*, *tim44*, *arg1*, *acta2* (*a-SMA*), and *col1a1*, were as described previously (27, 58). Other primers included *nr1h2,* left 5′-agctctgcctacatcgtggt-3′, right 5′-aagccttgtctccgcaca-3′; *nr1h3,* left 5′-cgcgacagttttggtagagg-3′, right 5′-ctccagccacaaggacatc-3′; and *abca1,* left 5′-atggagcagggaagaccac-3′, right 5′-gtaggccgtgccagaagtt-3′.

### Statistics.

Results are shown as mean ± SD. Statistical analyses were performed using a multiple unpaired 2-tailed Student’s *t* test for direct 2 group comparison or 2-way ANOVA for multiple group comparisons. All analyses were performed using GraphPad Prism. *P* < 0.05 (2-tailed) was considered statistically significant.

### Study approval.

The animal studies described were reviewed and approved by the UCLA IACUC).

## Author contributions

HZ, MN, HW, JZ, DJ, and YZ performed experiments and analyzed data; RWB, JWKW, WL, XW, and YZ designed experiments; YZ, HZ, and MN drafted the manuscript.

## Supplementary Material

Supplemental data

## Version 1 11/24/2022

In-Press Preview

## Version 2 01/10/2023

Electronic publication

## References

[1] Eltzschig HK, Eckle T (2011). Ischemia and reperfusion--from mechanism to translation. Nat Med.

[2] Zhai Y (2011). Liver ischemia and reperfusion injury: new insights into mechanisms of innate-adaptive immune-mediated tissue inflammation. Am J Transplant.

[3] Tsung A (2007). HMGB1 release induced by liver ischemia involves Toll-like receptor 4 dependent reactive oxygen species production and calcium-mediated signaling. J Exp Med.

[4] Tsung A (2005). The nuclear factor HMGB1 mediates hepatic injury after murine liver ischemia-reperfusion. J Exp Med.

[5] Zhai Y (2004). Cutting edge: TLR4 activation mediates liver ischemia/reperfusion inflammatory response via IFN regulatory factor 3-dependent MyD88-independent pathway. J Immunol.

[6] Bamboat ZM (2010). Toll-like receptor 9 inhibition confers protection from liver ischemia-reperfusion injury. Hepatology.

[7] Huang H (2011). Endogenous histones function as alarmins in sterile inflammatory liver injury through Toll-like receptor 9 in mice. Hepatology.

[8] Beurel E, Jope RS (2006). The paradoxical pro- and anti-apoptotic actions of GSK3 in the intrinsic and extrinsic apoptosis signaling pathways. Prog Neurobiol.

[9] Beurel E (2010). Innate and adaptive immune responses regulated by glycogen synthase kinase-3 (GSK3). Trends Immunol.

[10] Cohen P, Goedert M (2004). GSK3 inhibitors: development and therapeutic potential. Nat Rev Drug Discov.

[11] McManus EJ (2005). Role that phosphorylation of GSK3 plays in insulin and Wnt signalling defined by knockin analysis. EMBO J.

[12] Rayasam GV (2009). Glycogen synthase kinase 3: more than a namesake. Br J Pharmacol.

[13] Wang H (2011). Glycogen synthase kinase 3: a point of convergence for the host inflammatory response. Cytokine.

[14] Wu D, Pan W (2010). GSK3: a multifaceted kinase in Wnt signaling. Trends Biochem Sci.

[15] Hoeflich KP (2000). Requirement for glycogen synthase kinase-3beta in cell survival and NF-kappaB activation. Nature.

[16] MacAulay K (2007). Glycogen synthase kinase 3alpha-specific regulation of murine hepatic glycogen metabolism. Cell Metab.

[17] Suzuki T (2013). Inhibition of AMPK catabolic action by GSK3. Mol Cell.

[18] Martin M (2005). Toll-like receptor-mediated cytokine production is differentially regulated by glycogen synthase kinase 3. Nat Immunol.

[19] Beurel E (2015). Glycogen synthase kinase-3 (GSK3): regulation, actions, and diseases. Pharmacol Ther.

[20] Ren F (2011). Inhibition of glycogen synthase kinase 3 beta ameliorates liver ischemia reperfusion injury by way of an interleukin-10-mediated immune regulatory mechanism. Hepatology.

[21] Zhou H (2018). Glycogen synthase kinase 3β promotes liver innate immune activation by restraining AMP-activated protein kinase activation. J Hepatol.

[22] Ni M (2020). Isoform- and cell type-specific roles of glycogen synthase kinase 3 N-terminal serine phosphorylation in liver ischemia reperfusion injury. J Immunol.

[23] Shen XD (2002). CD154-CD40 T-cell costimulation pathway is required in the mechanism of hepatic ischemia/reperfusion injury, and its blockade facilitates and depends on heme oxygenase-1 mediated cytoprotection. Transplantation.

[24] Devey L (2009). Tissue-resident macrophages protect the liver from ischemia reperfusion injury via a heme oxygenase-1-dependent mechanism. Mole Ther.

[25] Ellett JD (2010). Murine Kupffer cells are protective in total hepatic ischemia/reperfusion injury with bowel congestion through IL-10. J Immunol.

[26] Yue S (2017). Prolonged ischemia triggers necrotic depletion of tissue-resident macrophages to facilitate inflammatory immune activation in liver ischemia reperfusion injury. J Immunol.

[27] Ni M (2021). T-cell immunoglobulin and mucin domain-containing protein-4 is critical for Kupffer cell homeostatic function in the activation and resolution of liver ischemia reperfusion injury. Hepatology.

[28] Sakai M (2019). Liver-derived signals sequentially reprogram myeloid enhancers to initiate and maintain Kupffer cell identity. Immunity.

[29] Bonnardel J (2019). Stellate cells, hepatocytes, and endothelial cells imprint the Kupffer cell identity on monocytes colonizing the liver macrophage niche. Immunity.

[30] Boada-Romero E (2020). The clearance of dead cells by efferocytosis. Nat Rev Mol Cell Biol.

[31] Snodgrass RG (2020). Efferocytosis potentiates the expression of arachidonate 15-lipoxygenase (ALOX15) in alternatively activated human macrophages through LXR activation. Cell Death Differ.

[32] Thorp E (2011). Shedding of the Mer tyrosine kinase receptor is mediated by ADAM17 protein through a pathway involving reactive oxygen species, protein kinase Cδ, and p38 mitogen-activated protein kinase (MAPK). J Biol Chem.

[33] Sather S (2007). A soluble form of the Mer receptor tyrosine kinase inhibits macrophage clearance of apoptotic cells and platelet aggregation. Blood.

[34] Cai B (2016). MerTK cleavage limits proresolving mediator biosynthesis and exacerbates tissue inflammation. Proc Natl Acad Sci U S A.

[35] Louwe PA (2021). Recruited macrophages that colonize the post-inflammatory peritoneal niche convert into functionally divergent resident cells. Nat Commun.

[36] Duffield JS (2005). Selective depletion of macrophages reveals distinct, opposing roles during liver injury and repair. J Clin Invest.

[37] Holt MP (2008). Identification and characterization of infiltrating macrophages in acetaminophen-induced liver injury. J Leukoc Biol.

[38] Triantafyllou E (2018). MerTK expressing hepatic macrophages promote the resolution of inflammation in acute liver failure. Gut.

[39] Zigmond E (2014). Infiltrating monocyte-derived macrophages and resident kupffer cells display different ontogeny and functions in acute liver injury. J Immunol.

[40] Dal-Secco D (2015). A dynamic spectrum of monocytes arising from the in situ reprogramming of CCR2+ monocytes at a site of sterile injury. J Exp Med.

[41] Schlegel M (2016). The neuroimmune guidance cue netrin-1 controls resolution programs and promotes liver regeneration. Hepatology.

[42] Guillot A, Tacke F (2019). Liver macrophages: old dogmas and new insights. Hepatol Commun.

[43] Krenkel O, Tacke F (2017). Liver macrophages in tissue homeostasis and disease. Nat Rev Immunol.

[44] Roszer T (2017). Transcriptional control of apoptotic cell clearance by macrophage nuclear receptors. Apoptosis.

[45] Korns D (2011). Modulation of macrophage efferocytosis in inflammation. Front Immunol.

[46] Zheng L, Conner SD (2018). Glycogen synthase kinase 3β inhibition enhances Notch1 recycling. Mol Biol Cell.

[47] Lu L (2018). Myeloid Notch1 deficiency activates the RhoA/ROCK pathway and aggravates hepatocellular damage in mouse ischemic livers. Hepatology.

[48] Zagorska A (2020). Differential regulation of hepatic physiology and injury by the TAM receptors Axl and Mer. Life Sci Alliance.

[49] Lafdil F (2009). Growth arrest-specific protein 6 deficiency impairs liver tissue repair after acute toxic hepatitis in mice. J Hepatol.

[50] Llacuna L (2010). Growth arrest-specific protein 6 is hepatoprotective against murine ischemia/reperfusion injury. Hepatology.

[51] Kim SY (2016). Liver X receptor and STAT1 cooperate downstream of Gas6/Mer to induce anti-inflammatory arginase 2 expression in macrophages. Sci Rep.

[52] Choi JY (2015). Mer signaling increases the abundance of the transcription factor LXR to promote the resolution of acute sterile inflammation. Sci Signal.

[53] Glaria E (2020). Integrating the roles of liver X receptors in inflammation and infection: mechanisms and outcomes. Curr Opin Pharmacol.

[54] Madenspacher JH (2020). Cholesterol 25-hydroxylase promotes efferocytosis and resolution of lung inflammation. JCI Insight.

[55] A–Gonzalez N (2009). Apoptotic cells promote their own clearance and immune tolerance through activation of the nuclear receptor LXR. Immunity.

[56] Sen P (2007). Apoptotic cells induce Mer tyrosine kinase-dependent blockade of NF-kappaB activation in dendritic cells. Blood.

[57] Nishi C (2019). MERTK tyrosine kinase receptor together with TIM4 phosphatidylserine receptor mediates distinct signal transduction pathways for efferocytosis and cell proliferation. J Biol Chem.

[58] Zhai Y (2008). CXCL10 regulates liver innate immune response against ischemia and reperfusion injury. Hepatology.

