# Supplementary material for: Gsk3β regulates the resolution of liver ischemia/reperfusion injury via MerTK
Source: JCI Insight. 2023 Jan 10;8(1):e151819. doi: 10.1172/jci.insight.151819 (PMC9870084; doi:10.1172/jci.insight.151819)
Supplement: Supplemental data [file jciinsight-8-151819-s045.pdf]

Supplement

1. Immunofluorescence staining of F4/80+ cells in sham or IR livers after the clodronate liposome (CL) treatment. CL was administered 48h prior to the onset of liver ischemia. Sham livers were harvest w/o liver IR at 0h, and IR livers at day 7 post reperfusion. Tissue sections were stained with fluorochrome-labeled anti-F4/80 and DAPI. F4/80+ Cells were quantitated by counting green cells under fluorescence microscope (x400). Average numbers of F4/80+ cells/field of different experiment groups were plotted.

Results indicate no differences in the numbers of F4/80+ macrophages in IR livers at day 7 post reperfusion between myeloid Gsk3β WT and KO mice.

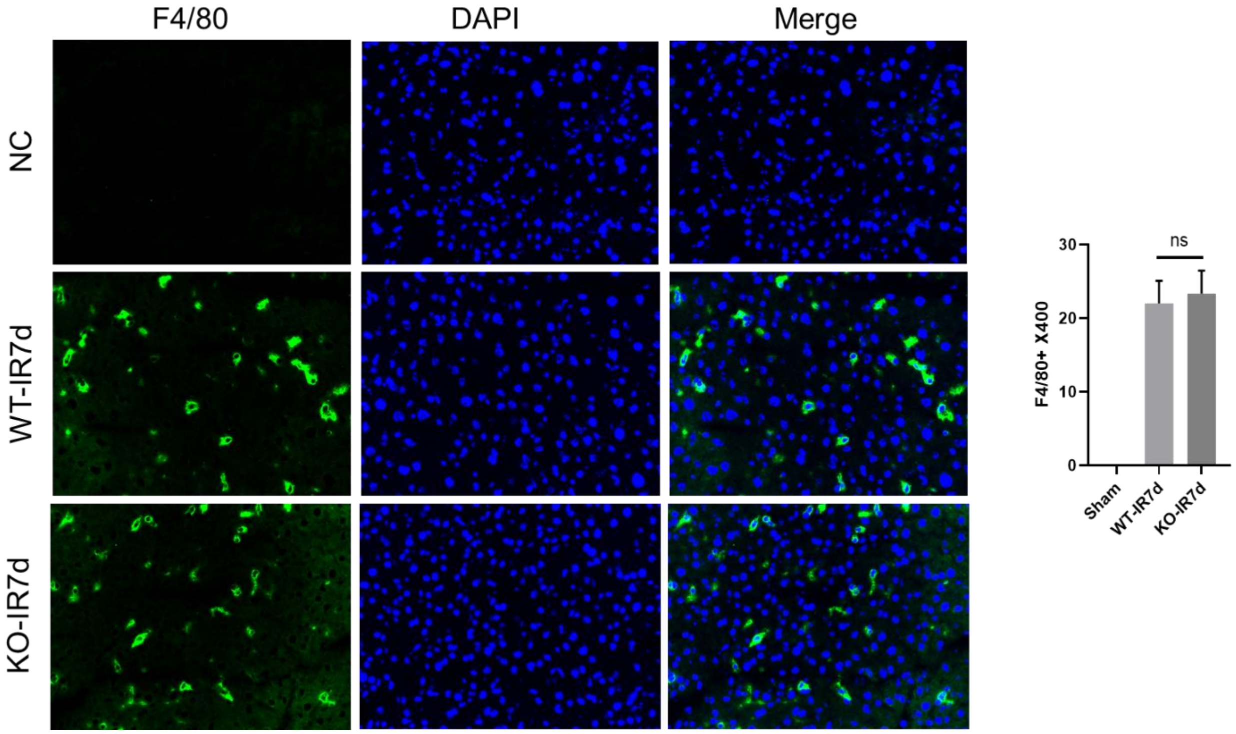

Sup Fig.1

2. Gsk3b regulates MerTK induction and efferocytosis in infiltrating macrophages (iMΦs) in LPS-induced peritonitis model. Groups of myeloid Gsk3β WT and KO mice were treated with either PBS or 15μg of LPS, i.p. Peritoneal cells were extracted by injecting 5ml cold PBS-EDTA (2mM) solution. One million cells were stained with CD11b-BV421, (Biolegend) TIM-4 PerCP, F4/80 BV650, MerTK-PE, Gr1-FITC (eBiosciences) and analyzed by BD LSRFortessa™ Cell Analyzer. (a) Kinetic changes of peritoneal infiltrating cells post LPS injection. Peritoneal cells were harvested from WT B6 mice after 24 and 48h post-PBS or LPS injection. Myeloid cell population were gated in FSC and SSC plots and analyzed for F4/80 and CD11b expression.

F4/80+CD11b+ (macrophages) and F4/80-CD11b+ (neutrophils) cell populations were further analyzed for Gr-1 and MerTK expression. (2) MerTK induction in iMΦs. Peritoneal cells were extracted at 48h post LPS injection. Myeloid cells were separated based on F4/80 and TIM-4 expressions. The iMΦs (F4/80^{+}TIM-4^{-}) were analyzed for MerTK and Gr-1 expression. (c) Efferocytosis assay of peritoneal iMΦs. Peritoneal cells were harvested at 48h post LPS injection. Adherent cells were collected and incubated with pHrodo labeled apoptotic thymocytes at 1 to 4 ratio in 12 well plate for 2h. Cells were washed and labeled with CD11b-FITC and TIM-4 PerCP. CD11b+TIM-4- cells (iMΦs) were gated and analyzed for pHrodo+ cells. Both density plot and histogram of % pHrodo+cells in iMΦs in were shown.

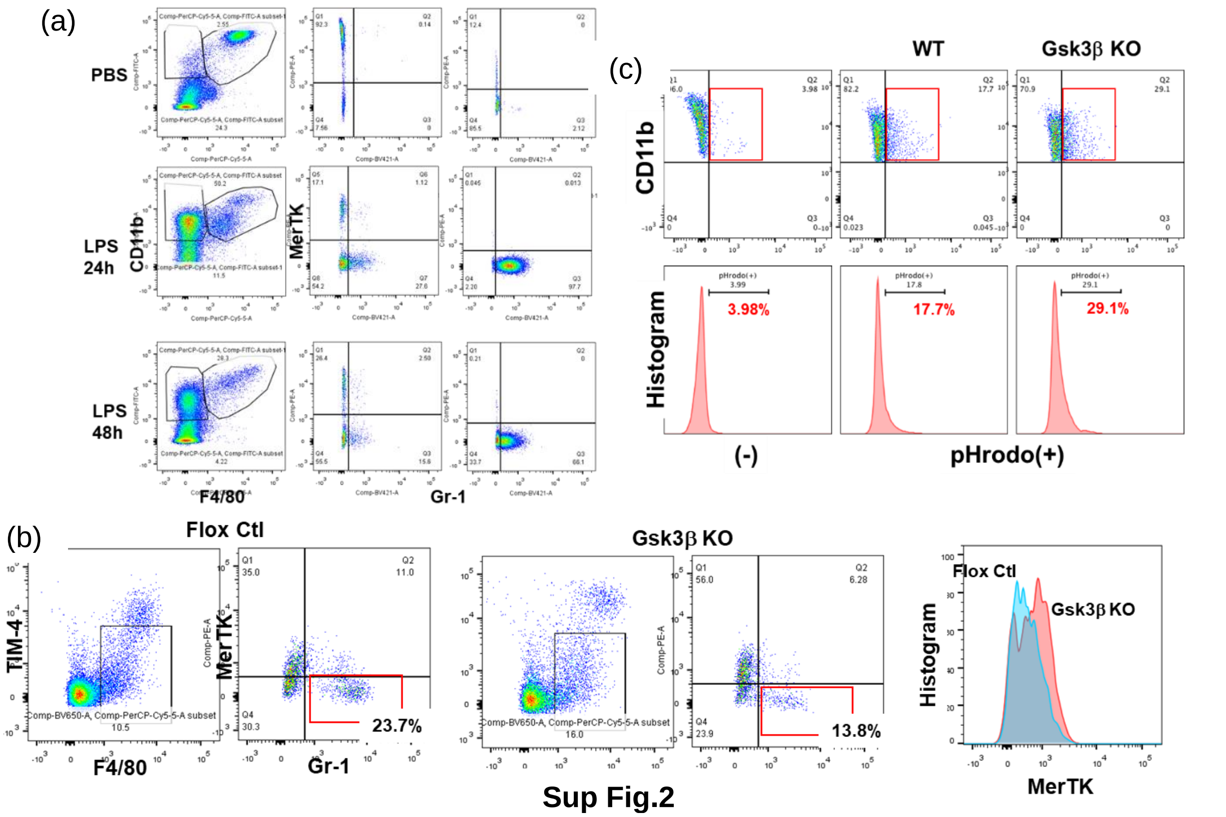

Sup Fig.2

Results: (a) The kinetic changes in peritoneal myeloid cell populations helped us to defined the resolution stage of the model. At 48h post LPS injection, Gr-1+ infiltrating (TIM-4-) macrophages were further decreased and MerTK+ cells increased from those at 24h. (b) MerTK induction was significantly higher and % of Gr-1+ was lower in Gsk3b deficient vs. WT iMΦs (TIM-4-F4/80+). (c) The efferocytosis function in Gsk3b deficient iMΦs was enhanced as compared with their WT counterparts, as measured in vitro using pHrodo-labeled apoptotic thymocytes as preys.

3. Impact of clodronate liposomes and diphtheria toxin treatments on liver macrophages post-IR. WT B6 or CD11b-DTR mice were treated with either PBS or CL or DT, as described in the Materials and Methods. Liver NPCs were isolated from IR livers at day 3 post reperfusion by in situ collagenase digestion. One million cells were stained with PerCP-Cy5.5-F4/80 and APC-CD11b and analyzed by BD LSRFortessa™ Cell Analyzer. Myeloid cell population was gated in FSC and SSC plot and analyzed for F4/80 and CD11b expression. F4/80^{+}CD11b^{low} cells represent KCs, F4/80^{+}CD11b^{high} cells represent iMϕ, and F4/80^{-}CD11b^{high} cells represent neutrophils.

Results: The CL treatment depleted KCs completely and also reduced iMϕ and neutrophils. The DT treatment did not reduced KCs, but significantly reduced iMϕ and neutrophils.

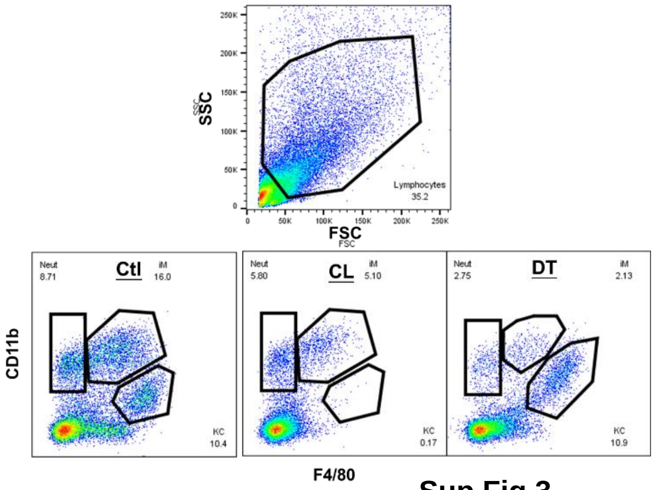

Sup Fig.3

4. MerTK expression in liver macrophages post IR. Liver NPCs were isolated from IR livers at day 3 post reperfusion of WT B6 mice. One million cells were stained with PerCP-Cy5.5-F4/80, FITC-CD11b and PE-MerTK and analyzed by BD LSRFortessa™ Cell Analyzer. Myeloid cell population was gated in FSC and SSC plot and analyzed for F4/80 and CD11b expression. MerTK expression levels were compared between KCs (F4/80^{+}CD11b^{low}) and iMϕs (F4/80^{+}CD11b^{high}). Neutrophils (F4/80^{-}CD11b^{high}) were included as control. MerTK histogram in liver macrophage subpopulations was shown.

Results: KCs express significantly higher levels of MerTK than iMϕs

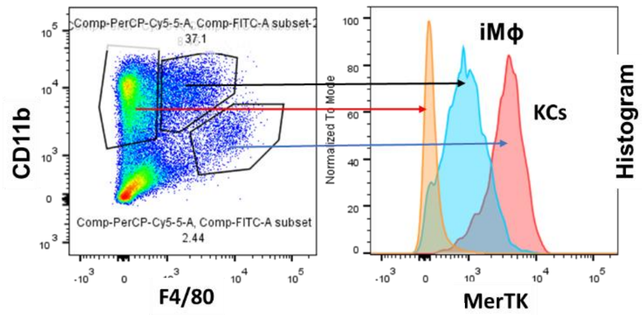

Sup Fig.4
